# Survival benefits of dose-dense early postoperative intraperitoneal chemotherapy in front-line therapy for advanced ovarian cancer: a randomised controlled study

**DOI:** 10.1038/s41416-019-0543-1

**Published:** 2019-08-06

**Authors:** Tingyan Shi, Rong Jiang, Hong Pu, Huijuan Yang, Dongsheng Tu, Zhiyuan Dai, Yunlang Cai, Yuqin Zhang, Xi Cheng, Huixun Jia, Ruiqin Tu, Huaying Wang, Jie Tang, Yuting Luan, Shumo Cai, Rongyu Zang, SGOG-OV/AICE Investigators

**Affiliations:** 1 Ovarian Cancer Program, Division of Gynecology Oncology, Department of Obstetrics and Gynecology, Zhongshan Hospital, Fudan University, Shanghai, China; 2 Department of Gynecologic Oncology, Fudan University Cancer Hospital, Shanghai, China; 3 Department of Obstetrics and Gynecology, Wuxi Caner Hospital, Jiangsu, China; 4 Department of Mathematics and Statistics, Queen’s University, Kingston, Canada; 5 Department of Obstetrics and Gynecology, Suzhou Municipal Hospital, Jiangsu, China; 6 Department of Obstetrics and Gynecology, Zhongda Hospital Southeast University, Jiangsu, China; 7 Clinical Statistics Center, Fudan University Cancer Hospital, Shanghai, China; 8 Clinical Statistics Center, Shanghai General Hospital, Shanghai, China

**Keywords:** Ovarian cancer, Randomized controlled trials

## Abstract

Dose-dense early postoperative intraperitoneal chemotherapy (DD-EPIC) significantly increased non-progression rate in advanced ovarian cancer (OC) patients. We report final overall survival (OS) results to further strengthen the efficacy of DD-EPIC in the front-line therapy. In this phase 2 trial, 218 patients with FIGO IIIC–IV OC were randomly allocated to receive DD-EPIC followed by intravenous (IV) chemotherapy (DD-EPIC group), or IV chemotherapy alone (IV group). The study was prespecified to detect differences in progression-free survival (PFS) and OS. At a median follow-up period of 69.1 months, the median OS was 67.5 and 46.3 months in the DD-EPIC and IV group, respectively. The probability rate of OS at 5 years was 61.0% with DD-EPIC, and 38.2% with IV (hazard ratio [HR] for death from OC, 0.70; 95% confidence interval [CI], 0.49–1.00). DD-EPIC was associated with a prolonged PFS compared with the IV group (the estimated rate of PFS at 5 years, 26.0% vs. 8.5%; HR for disease progression, 0.64; 95% CI, 0.47–0.86). DD-EPIC was associated with a longer OS than IV chemotherapy alone. It may be considered as a valuable option of the front-line therapy for advanced ovarian cancer.

**Trial registration:** ClinicalTrials.gov, NCT01669226 (date of registration: August 20, 2012).

## Background

To date, three randomised phase 3 clinical trials have demonstrated that intraperitoneal (IP) chemotherapy is an effective management for epithelial ovarian cancer after primary optimal debulking surgery.^1–3^ However, the fourth phase 3 trial, GOG252 that reduced the cisplatin dose from 100 mg/m^2^ to 75 mg/m^2^ neither showed a survival benefit in IP Carboplatin nor in IP Cisplatin,^4^ which highlights the controversy of IP chemotherapy in ovarian cancer. Our recent study reported disease progression delayed by additional dose-dense early postoperative intraperitoneal chemotherapy (DD-EPIC) with weekly cisplatin and etoposide (AICE trial) in the front-line setting.^5^ The primary endpoint of the AICE phase 2 trial showed a 28% increase in 12-month non-progression rate in ovarian cancer patients in favour of the DD-EPIC group [hazard ratio (HR) 0.48, 95% confidence interval (CI) 0.27–0.82, *P* = 0.005]. Despite the increased toxicity in the DD-EPIC group, most toxicities were acceptable, and the completion rate was much higher than that in GOG172, with the infection rate 11.6% in AICE vs. 16% in GOG172, and a completion rate of IP chemotherapy 90.6% vs. 42%, respectively.^2,5^ The mean inpatient cost in the DD-EPIC group was not much higher than that in the control group ($9338.2 vs. $7424.4).

The AICE trial was also designed to detect the improvement of progression-free and overall survival (OS). Here, we present the final OS and updated PFS data.

## Methods

### Trial design and procedure

The AICE study was an investigator-initiated multicentre, randomised, unblinded, controlled, phase 2 trial to assess the efficacy and safety of DD-EPIC in the front-line therapy of advanced ovarian cancer. The study design and the detailed inclusion and exclusion criteria, as well as the baseline characteristics and primary outcome results have been previously described.^5^ Briefly, eligible patients were aged 18–75 years; with stage IIIC and IV primary epithelial ovarian, fallopian tube or peritoneal cancer, excluding lymph node metastasis alone; an Eastern Cooperative Oncology Group (ECOG) performance status of 0–2; no more than three cycles of chemotherapy prior to surgery; with optimal (≤1 cm residual disease) debulking surgery. Randomisation was done using 1:1 allocation, and was performed after debulking surgery. DD-EPIC was defined to be started at 5–10 days after surgery, and no more than 14 days postoperative for those with bowel resection.

After randomisation, patients received either four doses of weekly DD-EPIC with cisplatin 50 mg/m^2^ and etoposide 100 mg/m^2^ followed by six cycles of IV carboplatin AUC 5 and paclitaxel 175 mg/m^2^ or docetaxel 60–75 mg/m^2^ every 3 weeks (the DD-EPIC group) or standard six cycles of IV carboplatin AUC 5 and paclitaxel 175 mg/m^2^ or docetaxel 60–75 mg/m^2^ every 3 weeks (the IV group).

Each patient was followed every 3 months over the first 5 years, and then every 6 months thereafter, during which physical examination, CA125 levels and radiological images (ultrasound, computed tomography or magnetic resonance imaging) were performed. Progression was defined by one or more of the following items: physical examination, elevated CA125 levels according to the Gynecologic Oncology Intergroup criteria and/or radiological images. Each progression event was confirmed by centre principle investigators.

### Statistical analysis

The trial was structured a priori to assess PFS and OS, as the secondary outcomes. PFS was defined as the time from randomisation to first recurrence/progression, or last follow-up, or death from ovarian cancer, whichever came first. The data regarding patients with no evidence of recurrence or death from ovarian cancer were censored at the date of last follow-up.

The comparisons and distributions of characteristics between the two groups and subgroups were conducted with the Chi-square or Student’s or Mann–Whitney *U* tests. Median survival was evaluated using the Kaplan–Meier method, and a log-rank test was used to compare survival between two randomised groups. Treatment effects were estimated by using the Cox regression model when proportional hazards could be assumed. Because there was no prespecified plan to stratify or adjust for multiple comparisons, a multivariable analysis was performed to evaluate efficacy outcomes with the adjustment for the important baseline confounders, such as FIGO stage, neoadjuvant chemotherapy and residual diseases. The previous analysis of 12-month non-progression rate has been conducted (two-sided α = 0.05).^5^ Here, prespecified PFS and OS analyses were performed after sufficient follow-up for the observation of 160 (80%) events of disease progression, or death from ovarian cancer, using a two-sided α of 0.05.

## Results

Censored on September 10, 2018 (3 years after the last patient enrolled), the median follow-up time was 69.1 months (interquartile range, 53.1–83.9). The median time to the first cycle of standard IV chemotherapy since primary surgery were 49 days and 15 days in the DD-EPIC and IV group, respectively (Student’s *t* test, *P* < 0.001), but the period of front-line chemotherapy was similar between the two groups, with only a 0.2-month increase in the DD-EPIC group.

**Fig. 1:**
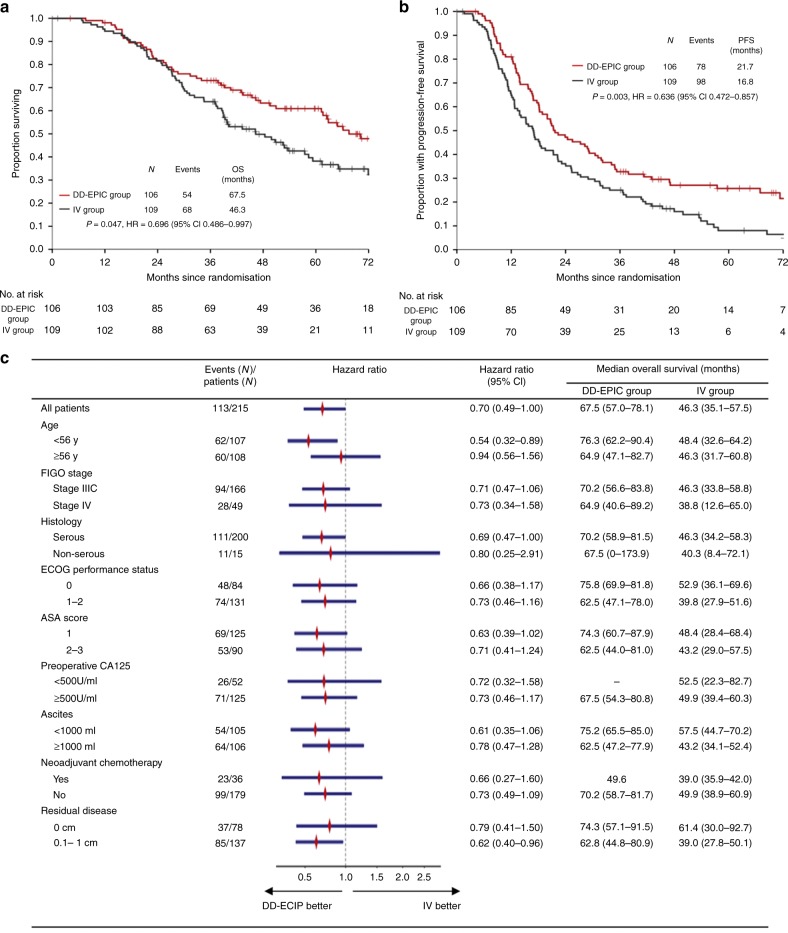
Treatment effect on survival. **a** Overall survival in all patients. **b** Progression-free survival in all patients. **c** Treatment effect on overall survival in major subgroups

Totally, 122 patients (56.3%) died from ovarian cancer: 54 (50.9%) of those in the DD-EPIC group and 68 (62.4%) of those in the IV group, respectively. The median overall survival was 67.5 (95% CI 57.0–78.1) months in the DD-EPIC group and 46.3 (95% CI 35.1–57.5) months in the IV group, a difference of 21.2 months. The probability rate of overall survival at 5 years was 61.0% with DD-EPIC and 38.2% with IV chemotherapy alone (HR for death from ovarian cancer, 0.70; 95% CI, 0.49–1.00; *P* = 0.047; Fig. 1a). Patients in the DD-EPIC group showed significantly prolonged PFS compared with those in the IV group (the estimated rate of PFS at 5 years, 26.0% vs. 8.5%; HR 0.64, 95% CI 0.47–0.86, *P* = 0.003; Fig. 1b). Similar findings were observed in the time to first and second subsequent anticancer therapies (Supplementary Figs. S1, S2). The survival benefit of DD-EPIC remained after the adjustment for FIGO stage, neoadjuvant chemotherapy and residual disease (Supplementary Table S1). Subgroup analyses of overall survival (Fig. 1c) and progression-free survival (Supplementary Table S2) showed that the benefit of DD-EPIC was consistent across most of the baseline risk factors and post hoc subgroups.

The maintenance therapy and the second-line therapy are listed in Supplementary Table S3. During the extended follow-up of overall survival, we did not observe any chemotherapeutic-related adverse events added.

## Discussion

Since the negative result of GOG252 with the reduction of the cisplatin dose from 100 mg/m^2^ to 75 mg/m^2^,^4^ IP chemotherapy in ovarian cancer has remained controversial, particularly in the improvement of study design. Recently, there were several novel study designs of IP chemotherapy, such as hyperthermic intraperitoneal chemotherapy (HIPEC),^6^ pressurised intraperitoneal aerosol chemotherapy (PIPAC),^7^ early postoperative Intraperitoneal chemotherapy (EPIC), amongst others. HIPEC demonstrated a survival benefit in patients who underwent neoadjuvant chemotherapy followed by interval cytoreduction.^6^ However, the effect of HIPEC is unclear in patients who underwent primary debulking surgery. PIPAC trial was a phase I study with intraperitoneal cisplatin and doxorubicin applied.^7^ Here, we present the final OS result of DD-EPIC in advanced ovarian cancer patients. With the median follow-up of 69.1 months, a remarkable overall survival benefit was recorded in favour of DD-EPIC with a 21.2-month improvement.

EPIC, as an approach of perioperative IP chemotherapy, has been used as an adjunct to oncologic surgery for peritoneal carcinomatosis, colorectal and gastric cancer, and other solid tumours.^8^ Goodman et al. indicated that EPIC could minimise non-uniform drug distribution, and eliminate residual cancer cell entrapment in post-operative fibrin deposits compared with the post-operative IP combined with IV therapy; and it is administered from post-operative day 1 with continued daily therapy for 5–7 days.^8^ Klaver et al. compared the two IP chemotherapy designs (HIPEC and EPIC) in colorectal peritoneal carcinomatosis. They found that both EPIC and HIPEC were effective in prolonging survival, but the beneficial effect of EPIC on survival seemed to be more pronounced than that of HIPEC.^9^ In the current trial, DD-EPIC was generally performed 4–7 days after surgery with continued weekly therapy for four doses. The addition of DD-EPIC, with only a median 0.2-month increase of the front-line therapy, did not obviously delay the start of standard IV chemotherapy. In total, there were 21.9% and 30.6% of patients identified to be platinum resistant with progression-free interval < 6 months in the DD-EPIC and IV group, respectively. It is reasonable to address that DD-EPIC might decrease platinum resistance and prolong non-progression interval.

Etoposide has previously been considered as a primary therapy for ovarian cancer.^10^ In a pharmacokinetic study of IP cisplatin and etoposide, the free (non-protein-bound) etoposide peritoneal exposure was 65-fold greater than that in plasma.^11^ Consistent with our previous study,^10^ IP chemotherapy of cisplatin and etoposide have also been reported to be effective in the primary treatment of ovarian cancer.^12^ There were no data about the usage of etoposide during the first-line therapy, however, we found some evidences from the second-line therapy, which indicated that etoposide might eliminate chemoresistant cancer cells. For example, recently, a combination therapy of apatinib and etoposide showed an impressive median objective response rate (ORR) of 54% (95% CI 36.6–71.2%) in patients with chemoresistant or chemorefractory recurrent ovarian cancer.^13^ In comparison, a previous report of apatinib alone in chemoresistant gastric cancer indicated that the ORR of apatinib alone was 6.4% and 13.0% for 850 mg once a day and for 425 mg twice a day, respectively,^14^ which is far less than the combined effect with etoposide in ovarian cancer. Therefore, it could be reasonable to address that ovarian cancer patients with chemoresistance may benefit more from etoposide rather than apatinib. This may partly explain why the platinum-resistant rate in the DD-EPIC group was much lower than that in the IV group (21.9% vs. 30.6%) in the current AICE trial. Following the overall survival data, the pattern of DD-EPIC combining cisplatin and etoposide may be more useful to decrease 12-month progression, thus improving PFS and OS.

Key points of the AICE study:

- Validation of more than 30 years’ experience with IP of cisplatin and etoposide in China.
- A high dose of cisplatin with total 200 mg/m^2^, obviously benefits those patients with age < 56 years old.

## Supplementary information

Supplementary Figures and Tables

## Data Availability

Each original data and Case Report Form for each patient used in the article can be accessed from the ethics committee of each participant hospital.
