# Supplementary material for: Survival benefits of dose-dense early postoperative intraperitoneal chemotherapy in front-line therapy for advanced ovarian cancer: a randomised controlled study
Source: Br J Cancer. 2019 Aug 6;121(5):425–8. doi: 10.1038/s41416-019-0543-1 (PMC6738091; doi:10.1038/s41416-019-0543-1)
Supplement: Supplementary file 1 — Supplementary Figures and Tables [file 41416_2019_543_MOESM1_ESM.doc]

**FIGURE LEGENDS**

**Figure 1**. Treatment effect on survival

A. Overall survival in all patients; B. Progression-free survival in all patients; C. Treatment effect on overall survival in major subgroups.

**Supplementary Figure S1.** Time to first subsequent anticancer therapy.

**Supplementary Figure S2.** Time to second subsequent anticancer therapy.

**Supplementary Figure S1. Time to first subsequent anticancer therapy.**

| | **No. at risk** |  |  |  |  |  |  |  | | --- | --- | --- | --- | --- | --- | --- | --- | | DD-EPIC group | 106 | 87 | 53 | 36 | 22 | 14 | 7 | | IV group | 109 | 74 | 40 | 26 | 14 | 6 | 4 | |
| --- | --- | --- | --- | --- | --- | --- | --- | --- | --- | --- | --- | --- | --- | --- | --- | --- | --- | --- | --- | --- | --- | --- | --- | --- |

**Supplementary Figure S2.** Time to second subsequent anticancer therapy.

| | **No. at risk** |  |  |  |  |  |  |  | | --- | --- | --- | --- | --- | --- | --- | --- | | DD-EPIC group | 102 | 97 | 76 | 50 | 34 | 23 | 11 | | IV group | 95 | 81 | 59 | 32 | 19 | 12 | 6 | |
| --- | --- | --- | --- | --- | --- | --- | --- | --- | --- | --- | --- | --- | --- | --- | --- | --- | --- | --- | --- | --- | --- | --- | --- | --- |

**Supplementary Table S1.** Efficacy outcome analyses according to randomized treatment.

|  | ***No. of evens (%)*** | | **Hazard ratio (95%CI)**  *vs. IV group* | ***P* value** |
| --- | --- | --- | --- | --- |
|  | DD-EPIC group  (*N* =106) | IV group  (*N* =109) |  |
| **Overall survival (OS)** |  |  |  |  |
| Unadjusted analysis | 54 (50·9) | 68 (62·4) | 0·70 (0·49-1.00) | 0·047 |
| Adjusted analysis* |  |  | 0·69 (0·48-1·00) | 0·048 |
| **Progression-free survival (PFS)** |  |  |  |  |
| Unadjusted analysis | 78 (73·6) | 98 (89·9) | 0·64 (0·47-0·86) | 0·003 |
| Adjusted analysis* |  |  | 0·65 (0·48-0·87) | 0·004 |
| **Time to first subsequent anticancer therapy (TFST)** | |  |  |  |
| Unadjusted analysis | 76 (71·7) | 97 (89·0) | 0·62 (0·46-0·83) | 0·002 |
| Adjusted analysis* |  |  | 0·63 (0·47-0·85) | 0·003 |
| **Time to second subsequent anticancer therapy (TSST)^#^** | |  |  |  |
| Unadjusted analysis | 61 (59·2) | 67 (70·5) | 0·66 (0·47-0·94) | 0·019 |
| Adjusted analysis* |  |  | 0·67 (0·47-0·95) | 0·024 |

*adjusted by FIGO stage, neoadjuvant chemotherapy, and residual disease.

^#^There were 103 and 95 patients have available follow up data of TSST in DD-EPIC and IV group respectively.

**Supplementary Table S2.** Treatment effect on progression-free survival in major subgroups

|  | Events(N)/  Patients(N) | Hazard ratio  (95% CI) | Median progression-free survival (months) | |
| --- | --- | --- | --- | --- |
|  |  |  | DD-EPIC group | IV group |
| All patients | 176/215 | 0.64 (0.47-0.86) | 21.7 (15.2-28.1) | 16.8 (13.3-20.3) |
| Age |  |  |  |  |
| <56yrs | 84/107 | 0.54 (0.35-0.84) | 28.8 (18.5-39.1) | 15.3 (10.6-20.0) |
| ≥56yrs | 92/108 | 0.77 (0.51-1.16) | 20.0 (15.5-24.4) | 17.0 (10.5-23.5) |
| FIGO stage |  |  |  |  |
| Stage IIIC | 137/166 | 0.62 (0.45-0.88) | 23.9 (16.7-31.1) | 17.1 (13.1-21.1) |
| Stage IV | 39/49 | 0.67 (0.35-1.29) | 18.2 (8.8-27.5) | 15.3 (8.2-22.3) |
| Histology |  |  |  |  |
| Serous | 164/200 | 0.64 (0.47-0.87) | 21.3 (14.6-28.1) | 16.8 (13.8-19.8) |
| Non Serous | 12/15 | 0.62 (0.20-1.97) | 24.4 (8.0-40.9) | 12.7 (6.8-18.6) |
| ECOG performance status |  |  |  |  |
| 0 | 68/84 | 0.61 (0.38-0.98) | 28.8 (12.3-45.3) | 15.3 (10.9-19.7) |
| 1-2 | 108/131 | 0.67 (0.46-0.99) | 20.0 (15.2-24.7) | 17.0 (12.5-21.6) |
| ASA Score |  |  |  |  |
| 1 | 96/125 | 0.55 (0.36-0.83) | 29.2 (14.6-43.8) | 17.3 (13.8-20.7) |
| 2-3 | 80/90 | 0.80 (0.51-1.24) | 18.2 (13.2-23.2) | 15.8 (10.4-21.2) |
| Preoperative CA125 |  |  |  |  |
| <500U/ml | 38/52 | 0.47 (0.25-0.92) | 32.2 (8.6-55.7) | 18.3 (10.4-26.1) |
| ≥500U/ml | 103/125 | 0.71 (0.48-1.05) | 21.7 (11.3-32.0) | 17.0 (14.0-19.9) |
| Ascites |  |  |  |  |
| <1000ml | 82/105 | 0.49 (0.31-0.77) | 27.3 (15.1-39.4) | 17.0 (13.8-20.1) |
| ≥1000ml | 90/106 | 0.82 (0.54-1.24) | 20.0 (14.1-25.8) | 15.3 (5.4-25.2) |
| Neoadjuvant chemotherapy | |  |  |  |
| Yes | 28/36 | 0.36 (0.15-0.87) | 18.1 | 13.8 (8.8-18.9) |
| No | 148/179 | 0.71 (0.51-0.98) | 21.7 (16.5-26.8) | 17.0 (14.2-19.9) |
| Residual disease (PFS) |  |  |  |  |
| 0 cm | 58/78 | 0.47 (0.28-0.80) | 34.8 (3.1-66.5) | 16.8 (13.7-20.0) |
| 0.1- 1 cm | 118/137 | 0.76 (0.53-1.09) | 18.2 (14.3-22.1) | 15.8 (10.2-21.4) |

**Supplementary Table S3.** Second-line treatment after first recurrence

| **Treatment** | **DD-EPIC group** | **IV group** | **Total** | ***P* value** |
| --- | --- | --- | --- | --- |
| Secondary cytoreduction | 21 (26.9%) | 15 (15.3%) | 36 (20.5%) | 0.073^a^ |
| Chemotherapy alone | 53 (67.9%) | 81 (82.7%) | 134 (76.1%) |  |
| No treatment | 4 (5.1%) | 2 (2.0%) | 6 (3.4%) |  |
| Endocrine drugs | 0 | 0 | 0 | NA |
| Olaparib maintenance | 4 (5.1%)^b^ | 2 (2.0%)^c^ | 6 (3.4%) | NA |
| SOLO2 trial of Olaparib | 1 (1.3%) | 0 | 1 (0.6%)^d^ | NA |

^a^ There was no significant difference between the two groups in second-line treatment after first recurrence (among secondary cytoreduction, chemotherapy alone, and no treatment; Chi- square test, *P*=0.073).

^b^ Four patients received Olaparib maintenance therapy during the second-line therapy, however, three of whom participated in the L-MOCA single arm trial of Olaparib on August 2018.

^c^ Two patients participated in the L-MOCA single arm trial of Olaparib maintenance therapy on August 2018.

^d^ One patient participated in the SOLO2/ENGOT-Ov21 double-blind, randomized, placebo-controlled, phase 3 trial after second-line therapy, and the therapeutics was still in blind.
